# Parthenolide Attenuates Sepsis-Induced Acute Kidney Injury in Rats by Reducing Inflammation

**DOI:** 10.1155/2023/8759766

**Published:** 2023-01-06

**Authors:** Di-Wen Shou, Yi-Rong Li, Xiu-Juan Xu, Mu-Hua Dai, Wei Zhang, Xue Yang, Yue-Xing Tu

**Affiliations:** ^1^The First Affiliated Hospital of Zhejiang Chinese Medical University (Zhejiang Provincial Hospital of Traditional Chinese Medicine), Hangzhou 310012, China; ^2^Department of Critical Care Medicine, Tongde Hospital of Zhejiang Province, Hangzhou 310012, China; ^3^Emergency and Critical Care Center, Intensive Care Unit, Zhejiang Provincial People's Hospital (Affiliated People's Hospital Hangzhou Medical College), Hangzhou 310014, China; ^4^Clinical Research Institute, Zhejiang Provincial People's Hospital, Affiliated People's Hospital, Hangzhou Medical College, Hangzhou 310014, China

## Abstract

**Background:**

Sepsis is a common complication of severe trauma, burns, infection, or major surgery. This disease-related end-organ dysfunction results from systemic inflammatory response syndrome (SIRS). Acute kidney damage (AKI), also known as acute renal failure, is one of the most frequent and serious sequelae of sepsis. Nuclear transcription factor-*κ*B (NF-*κ*B) regulates the transcription of inflammation-related genes and operates as a mediator in the immune system. While parthenolide (PTL) has been reported to prevent harmful inflammatory reactions, its effects on sepsis-associated AKI are unknown. The current study investigates the effects of PTL in sepsis-associated AKI using cell and cecal ligation and puncture (CLP) models.

**Methods:**

Lipopolysaccharide (LPS)-stimulated rat glomerular mesangial cells were treated with 10 *μ*M PTL. Inflammatory mediators, including TNF-*α*, IL-6, and IL-1*β*, in the culture supernatants were measured by ELISA, and NF-*κ*B levels were assessed by qPCR. After the generation of the septic CLP model, rats were intraperitoneally injected with 500 g/kg PTL and were euthanized after 72 h. Serum and kidney samples were analyzed.

**Results:**

TNF-*α*, IL-1*β*, and IL-6 levels were elevated after LPS treatment of rat glomerular mesangial cells (*p*=0.004, *p*=0.002, and *p*=0.004, respectively) but were significantly reduced in the PTL treatment group (*p* ≤ 0.001, *p*=0.01, and *p* ≤ 0.001). NF-*κ*B p65 levels were also increased after LPS treatment in this group and were reduced in the PTL treatment group. PTL treatment also reduced kidney damage after CLP induction, as shown by histological analysis and reductions in the levels of BUN, Cre, KIM-1, and NAGL. CLP-induced kidney inflammation together with increased levels of proinflammatory cytokines and inflammatory-related proteins. The elevated levels of renal TNF-*α*, IL-6, and IL-1*β* were downregulated after PTL treatment. The PTL treatment also reduced the CLP-induced activation of NF-*κ*B p65 in the damaged kidneys.

**Conclusion:**

PTL reduced inflammation induced by CLP-induced AKI in rat models and LPS-induced damage to glomerular mesangial cells by suppressing NF-*κ*B signaling.

## 1. Introduction

Extreme injuries, burns, infections, or extensive surgery often result in sepsis. Illness-induced end-organ damage is due to a systemic inflammatory response syndrome (SIRS). When septic shock develops, it causes numerous organ dysfunction syndromes, which have a mortality rate between 30% and 70% [1]. There is a wide variety of pathogens and a complicated set of pathways that may lead to sepsis. Epigenetic and transcriptional regulation, neuroendocrine-immune network problems, aberrant coagulation, tissue and organ damage, inflammatory and metabolic damage, and the impact of microbial toxins on the host are all examples [1–3]. Therefore, it has been difficult to find an effective medicine for the prevention and treatment of sepsis that simultaneously has a favorable safety profile and addresses all of these concerns.

There is a close relationship between local renal inflammation and acute renal injury in sepsis. It is well known that endotoxins and infections penetrate the body, causing the production of several inflammatory mediators and the upregulation of serum inflammatory markers such as IL-2, IL-6, TNF-*α*, and CRP [3]. Additionally, they disturb the immune and anti-inflammatory systems and intestinal flora, leading to coagulation disorders and organ dysfunction [4]. Sepsis has been classified as an “exogenous febrile disease” in traditional Chinese medicine (TCM) [5], which is important in treating sepsis with its unique theoretical system.

Regarding sepsis treatment, TCM has accumulated a wealth of experience. Lu et al. reported that TCM clears away heat and toxins, kills bacteria, and has a pivotal role in regulating immunity and promoting the reconstruction of the neuroendocrine system [6]. A growing number of studies revealed that TCM may enhance outcomes in the sepsis model by regulating inflammation levels [7–13]. Meanwhile, TCM's efficacy in treating sepsis was also validated in a meta-analysis of 10 randomized controlled studies conducted by Liang et al. [14]. These studies showed that TCM may play an important role in sepsis treatment.

*Tanacetum vulgare* L. is an herb that has been classified in traditional Chinese herbal medicine. Importantly, it has analgesic, antibacterial, and antitumor effects. Moreover, it can be used for fever, migraines, and joint pains. The chrysanthemum has a wide application against inflammatory diseases such as fever and arthritis due to its active anti-inflammatory ingredients. Various medicinal components can be extracted from wormwood, among which sesquiterpene lactone has proven to be the component with the best pharmacological activity. A sesquiterpene lactone called *parthenolide* (PTL) is extracted from the perennial plant feverfew and showed anti-inflammatory properties [15–17]. These effects are widely considered to be the results of the inhibitory effect on transcription factors of the NF-*κ*B family [18–20]. Activation of NF-*κ*B may boost IL-2, IL-1*β*, and TNF-*α* transcription, expression, and release, which in turn can activate NF-*κ*B again [21, 22]. The NF-*κ*B could promote the release of IL-6 and other inflammatory cytokines, which would then further amplify the initial inflammatory signal and trigger the “cascade reaction” of inflammatory mediators. As a result, it can provoke damage to the kidney and other organs, which plays a vital role in the development of sepsis and renal impairment [23]. PTL has been shown to inhibit platelet phospholipase A 2,5-lipoxygenase directly and induce epoxygenase, thereby assisting in the prevention of inflammation by NF-*κ*B inhibition [24, 25]. By preventing the synthesis of proinflammatory mediators, including nitric oxide, PGE, and TNF-*α*, PTL inhibits the production of TNF-*α*, IFN-*γ* IL-2, and IL-4 in human peripheral blood, according to other research studies [15, 26].

In this study, after the sepsis model was established in rats using the CLP technique, we investigated the pathological alterations in the renal tissues and the way they affected renal function [27]. Subsequently, after that, we kept a record of the differences between rats treated with PTL and those not, including the marker of inflammation and the pathway profile. After administering PTL treatment, we further stimulated rat renal mesangial cells with LPS and observed the cellular inflammatory response. The objective of the present study is to examine PTL's protective role in infected rats.

## 2. Materials and Methods

### 2.1. Cells

Rat mesangial cell lines (HBZY-1) were stored in laboratory conditions. First, 37°C in a humid environment with 5% CO_2_ was used to cultivate HBZY-1 cell lines in DMEM High Glucose (ThermoFisher, Waltham, MA, USA) supplemented with 10% FBS. The cells were then plated onto 6- or 12-well dishes measuring about 10 cm in diameter. The HBZY-1 cell lines were grown in serum-free DMEM for 24 hours to synchronize cell growth before being employed for tests after they had achieved 60–70% confluence.

### 2.2. Parthenolide

PTL derives from the medicinal plant feverfew and is a sesquiterpene lactone. MedChemExpress (MCE, CAS No. 20554-84-1) was used to acquire the PTL and LPS, and both were kept at room temperature after they were purchased.

### 2.3. MTT Assay

MTT (Sangon Biotech, Shanghai, China) assays were used to assess cell growth. Cells were seeded in 96-well plates and treated with 20 *μ*g/ml of LPS and varying doses of PTL over 48 h. Each condition was assessed in triplicate. MTT was added to the cells and incubated at 37°C for 30 min, according to the instructions provided with the kit. After the addition of DMSO (Sangon Biotech), absorbances were read at 450 nm in a microplate reader.

### 2.4. Animal Experiments

The experimental animal center of Zhejiang Provincial People's Hospital provided fifty male Sprague-Dawley rats (weighing 150–200 g), which were obtained and kept in a room with a controlled temperature of 21–25°C and a 12-hour light-dark cycle. Rats were randomly divided into three groups of twenty. The first group served as a comparison group since they were the sham-operation group of rats. The second set of rats was used to study septic conditions; they were designated as “CLP rats.” The rats in the third group (CLP + PTL) were used as septic models through CLP and then received a 500 g/kg PTL intraperitoneal injection two hours later. After waiting 24 hours, a vein in the inner canthal orbit was used to draw blood. Formalin was used to fix the kidney samples, both fixed and frozen. In order to determine the percentage of rats that made it up to day 7, we utilized 15 rats from each group. Both the Zhejiang Provincial People's Hospital and the People's Hospital at Hangzhou Medical University had their respective research ethics committees approve this work (Hangzhou, China).

### 2.5. ELISA

Following collection, blood samples were spun at 3,000 g for 10 minutes to remove clotting factors in anticoagulant tubes containing ethylenediaminetetraacetic acid (EDTA). To identify cytokines and indicators of renal damage by ELISA within three days, the supernatant (serum) was isolated and kept at 80°C. Instead, cytokines were detected using an ELISA on the supernatant recovered after centrifuging glomerular mesangial cells at 6,000 g for 5 minutes. We evaluated the serum concentrations of important cytokines in accordance with the guidelines provided by the ELISA kits. At 450 nm, the absorbance values were recorded. Concentrations of certain cytokines were determined after standard curves were established.

### 2.6. qPCR Analysis

For each biological sample, total RNA was extracted using RNA Easy Fast (TIANGEN, Beijing, China), and the RNA concentration and absorbance ratios (A260/280 and A260/230) were determined using a Nanodrop 2000 Spectrophotometer (NanoDrop Technologies, Japan). PrimeScriptTM RT reagent Kit (Takara, Beijing, China) was used to convert 1000 ng of RNA from each sample into cDNA in a final volume of 20 *μ*l. The concentration of the latter was then adjusted to 100 ng/*μ*l so that it could be evaluated by spectrophotometric analysis. After that, qPCR using TB Green® Premix Ex Taq™ was carried out (Takara, Beijing, China). Each reaction required a total volume of 20 L. A hot start cycle of 95°C for 3 minutes was programmed into the thermocycler, followed by 32 cycles of 95°C for 10 seconds and 59°C for 30 seconds. After each amplification, the purity of the final product was confirmed by analyzing the melting curve.

### 2.7. Western Blot Analysis

Dissected and homogenized in a radioimmunoprecipitation assay (RIPA) lysis buffer were rat kidney cortexes or cells (P0013B, Beyotime Biotechnology, Haimen, China). The quantities of proteins were determined using a bicinchoninic acid protein assay kit (Beyotime, Haimen, China) from the collected supernatant after centrifugation at 13,000 rpm for 15 minutes at 4°C. As a benchmark for protein content, bovine serum albumin was employed. Protein lysates were loaded in equal volumes onto a 10%–13% SDS-PAGE gel and transferred to a polyvinylidene difluoride membrane (0.2 *μ*m, Bio-Rad) for protein blotting. The membranes were treated with the appropriate primary antibodies at 4°C overnight after being blocked for 1 hour at room temperature with 5% BSA (w/v) in Tris-buffered saline with 0.1% Tween-20 (TBS-T). Following three 5-minute TBS-T washes, the membranes were incubated with horseradish peroxidase-labeled goat antimouse IgG (1 : 2000; Biosynthesis Biotechnology, Beijing, China) or goat antirabbit IgG (1 : 2000; Biosynthesis Biotechnology, Beijing, China) for 1 hour. The immunoblots may be seen using Bio-Rad ChemiDoc MP and Immobilon Western Chemiluminescent HRP Substrate from Millipore in Billerica, Massachusetts.

### 2.8. BUN and Serum Creatinine (Scr) Evaluation

Scr and BUN kits were bought from Nanjing Jiancheng Bioengineering Institute (Nanjing, China), and a biochemical analyzer automatically checked them (TC6010L, Tecom Science Corporation, Jiangxi, China). The picric acid technique was used to test Scr levels, whereas the urease method was used to measure BUN levels.

### 2.9. Renal Histological Examination

The kidney tissues were embedded in paraffin with a slice thickness of 4 m and fixed in 10% neutral buffered formalin. Deparaffinized and rehydrated kidney slices were stained with either periodic acid-Schiff or hematoxylin and eosin. Light microscopy was used to magnify the cells by 200x or 400x. In the semiquantitative analysis for the examination of the morphological alterations, each group included at least three samples, from which two sections at 200x magnification and 10 fields were selected at random. The proportion of renal tubules that were wounded or damaged, as shown by tubular lysis, dilatation, disruption, and cast formation, served as a measure of the histological alterations.

### 2.10. Immunohistochemical Staining

For the renal histology investigation, kidney samples were fixed as instructed. The parts were baked for two hours at 70°C. The antigen was recovered by deparaffinizing the slides in xylene, rehydrating them in an ethanol concentration gradient, and then boiling them for three minutes in 1 mM TE buffer at high pressure. Then, endogenous peroxidase activity was inhibited by blocking the area with 3% hydrogen peroxide for 15 minutes to reduce any residual nonspecific staining. Then, for 20 minutes, we added 10% goat nonimmune serum from Invitrogen in Carlsbad, California. According to the experimental protocol, rabbit polyclonal antibodies against human IL-1*β*, TNF-*α*, and IL-6 were used to stain tissue microarrays (dilution, 1 : 400; Santa Cruz Biotechnology, Inc., Dallas, TX, USA; cat. no. Sc-15405). This was done overnight at 4°C, and then the sections were incubated with the biotin-labeled secondary antibody (Invitrogen, Carlsbad, CA). The same sections were treated at room temperature for 15 minutes with HRP-conjugated streptavidin from Invitrogen in Carlsbad, California. Then, using the DAB Substrate Kit, color development was carried out (Dako, Glostrup, Denmark). After being counterstained with hematoxylin, the slices were dried, cleaned, and mounted.

### 2.11. Statistical Analysis

Unless otherwise specified, all tests were performed in triplicate. This information is displayed as mean ± SD. One-way analysis of variance was used to compare several groups, and then the Student–Newman–Keuls test was used to determine statistical significance. The two-tailed *t*-test was used to compare the groups, and a significance level of *p* < 0.05 was set.

## 3. Results

### 3.1. PTL Treatment Alleviated the LPS-Induced Decrease in Cell Viability In Vitro

**Figure 1:**
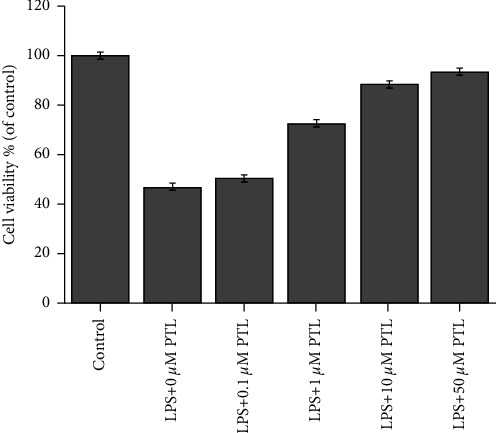
Effect of PTL on the LPS-induced proliferation of glomerular mesangial cells determined by MTT assay.

In order to determine the optimal concentration for PTL induction, we designed a series of PTL concentrations to treat glomerular mesangial cells with or without LPS induction. Glomerular mesangial cells were planted into 96-well plates, in which 20 *μ*g/ml LPS and different concentrations of PTL were added after adherence. After 24 hours, along with the increase in PTL concentrations, the survival rate increased gradually (Figure 1). With a PTL concentration of 10 *μ*M, the survival rate of the cells was restored to approximately 90%, so a 10 *μ*M concentration of PTL was selected for the subsequent experiments.

### 3.2. PTL Treatment In Vitro Reduced LPS-Induced Inflammatory Response

**Figure 2:**
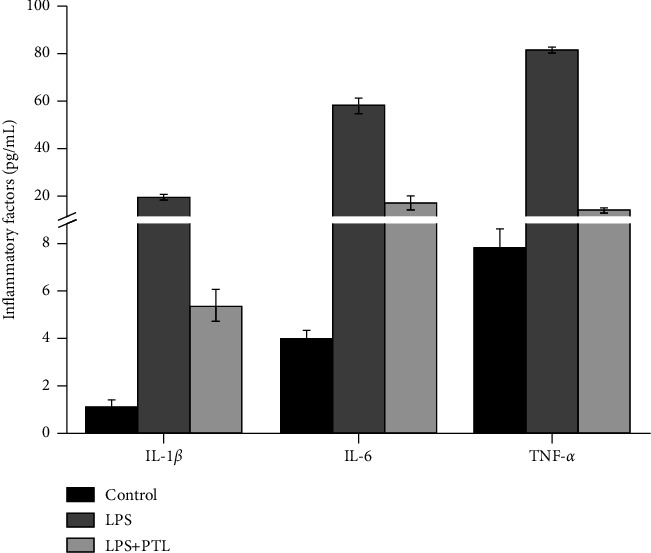
Effects of PTL on LPS-induced glomerular mesangial cell cytokines determined by ELISA.

To explore the effect of PTL on cytokines secreted by LPS-induced glomerular mesangial cells, the cells were planted as described above and then treated with 10 *μ*M PTL. The purification and detection of the supernatant of glomerular mesangial cells were performed after 24 hours.  Figure 2 illustrates the expression of IL-1*β*, IL-6, and TNF-*α*, detected by ELISA. Interestingly, the data indicated that IL-1*β*, IL-6, and TNF-*α* were all expressed at significantly higher levels after LPS stimulation (*p*=0.004, *p*=0.002, and *p*=0.004, respectively). By contrast, PTL attenuated the LPS-induced inflammatory factors expression (*p* ≤ 0.001, *p*=0.01, and *p* ≤ 0.001).

### 3.3. PTL Treatment Reduced NF-*κ*B/p65 Level Induced by LPS of In Vitro

**Figure 3:**
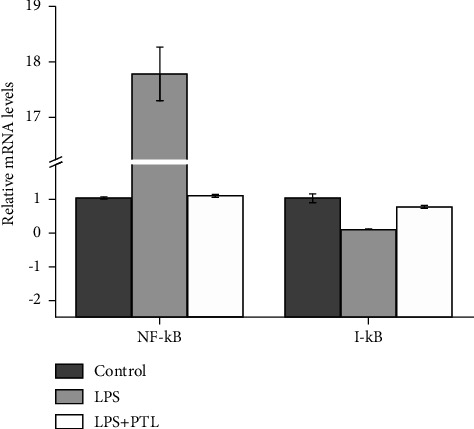
Expression of the NF-*κ*B-related LPS-induced genes on glomerular mesangial cells during PTL treatment. The values are represented as mean ± SD from three independent samples.

Here, 6-well plates were used for cell seeding. After 24 hours, total RNA of glomerular mesangial cells was isolated, and NF-*κ*B p65 expression was identified using the qPCR method (Figure 3). The NF-*κ*B p65 expression was found to be considerably upregulated induced by LPS (*p* ≤ 0.001). A decrease in p65 expression was downregulated after PTL treatment (*p* ≤ 0.001). The I-*κ*B expression exhibited a reversed tendency compared to NF-*κ*B. We detected the I-*κ*B enzyme's role in the NF-*κ*B signaling pathway. The LPS-induced I-*κ*B expression was downregulated (*p*=0.010). In contrast, the PTL induced the upregulation of I-*κ*B expression (*p*=0.005).

### 3.4. PTL Treatment Improved the Survival Rate

**Figure 4:**
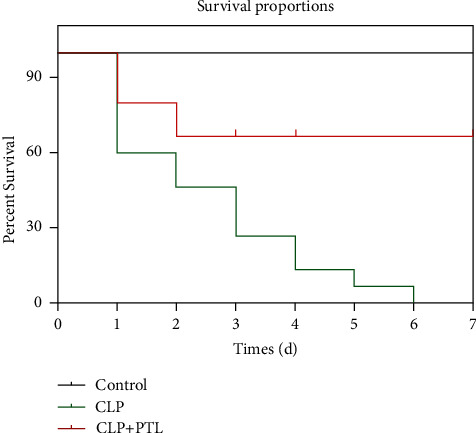
Rats that received PTL therapy had a considerably higher survival rate after undergoing the CLP procedure. For seven days, the mortality of the control, septic, and PTL (500 *μ*g/kg)-treated septic rats was observed twice daily. PTL was administered to the rats right after following the CLP procedure. The number of rats in each group was 15. The Kaplan–Meier analysis and the log-rank test were used to assess the survival rate.

The survival rate of the rats was also detected. There were three groups of animals: the control group, the CLP group, and the CLP + PTL group (*n* = 10). A CLP process was performed on the rats, which is shown in Figure 3. There was a drop in the survival rate after one day, followed by a 50% drop after two days. By the 7^th^ day, no rat in the CLP group was alive. As for the PTL group, a drop in the survival rate was observed in the septic rats after two days of treatment. When compared to the CLP group, however, the survival rate was raised by 60% by the 7^th^ day (Figure 4). According to the statistical analysis, this difference was very significant (*p* ≤ 0.001). The results strongly suggested that PTL treatment can drastically increase the survival proportion of septic rats.

### 3.5. PTL Protected Septic Rats from Renal Injury

**Figure 5:**
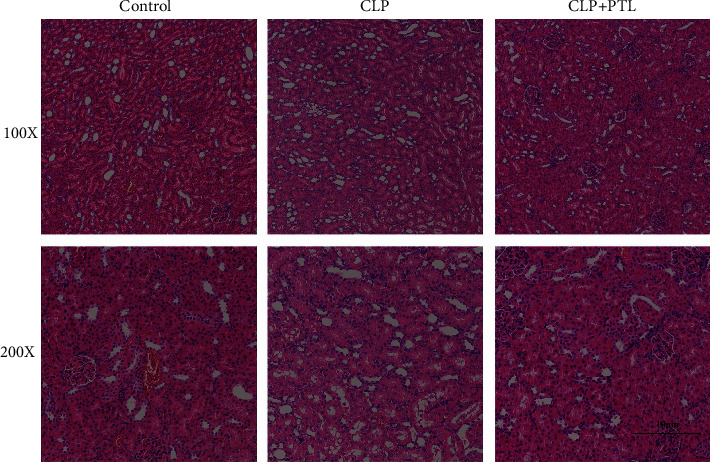
PTL's influence on renal damage in septic rats. Using a HE staining experiment (at 100x and 200x magnification), we were able to observe the histological alterations that CLP caused in the kidney tissues and calculate the renal damage score. In both the control and PTL groups, tubules were not damaged. Critical tubular injury was triggered by CLP. After being given CLP, renal tubular damage was healed by PTL. There is a 10 *µ*m scale bar there for perspective.

Histopathological alterations as a result of the CLP technique and PTL therapy are shown in Figure 5. All of the rats subjected to the CLP process had elevated levels of sepsis and renal damage in comparison to the control group. Increased inflammatory infiltrates, obvious oedema, more hemorrhagic regions in the interstitial, larger glomeruli, constricted renal tubules, and degraded epithelium were seen in the CLP group, thus suggesting that the CLP procedure induced sepsis in rats and caused substantial kidney injury in rats. However, compared to the CLP group, the renal structure in the PTL group did not show significant changes associated with injury. These results showed that PTL therapy protected the kidneys of septic rats.

**Figure 6:**
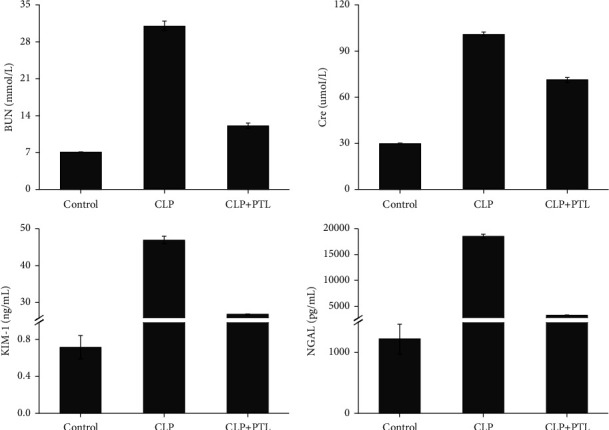
PTL reduced kidney damage brought on by CLP in vivo. BUN, Cre, KIM-1, and NGAL concentrations in various groups' peripheral blood. Each statistic is a mean ± SD value.

Furthermore, we also determined whether the PTL influenced the response of the kidney to damage. Some indicators of renal damage were tracked in the rats, including BUN, Cre, KIM-1, and NGAL. Increased levels of BUN (*p* ≤ 0.001), Cre (*p* ≤ 0.001), KIM-1 (*p* ≤ 0.001), and NGAL (*p* ≤ 0.001) were seen in CLP-treated rats. Injury to renal function is a warning sign (Figure 6). PTL therapy suppressed the increases in BUN (*p* ≤ 0.001), Cre (*p* ≤ 0.001), KIM-1 (*p* ≤ 0.001), and NGAL (*p* ≤ 0.001) compared to the CLP model.

### 3.6. PTL Alleviated Inflammation and Preserved Kidney Function in Rats

**Figure 7:**
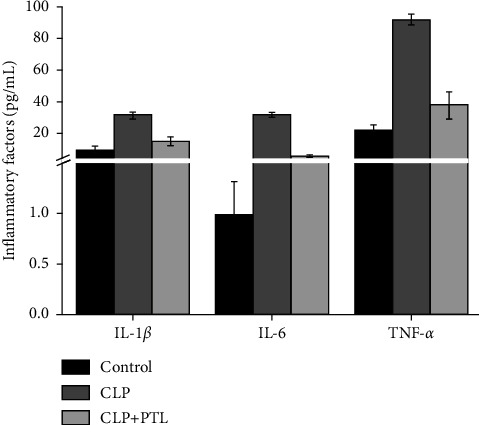
The in vivo release of peripheral blood cytokines by CLP was significantly decreased by PTL. TNF-*α*, IL-1*β*, and IL-6 levels in kidney tissues were measured using ELISA. The outcomes shown here are typical of at least three other trials. Every value is mean ± SD.

Our next step was to evaluate the PTL effect on the damaged kidney, and we assessed IL-1*β*, TNF-*α*, and IL-6 concentrations in peripheral blood (Figure 7). The results showed that proinflammatory cytokines IL-1*β* (*p*=0.002), TNF-*α* (*p* ≤ 0.001), and IL-6 (*p*=0.002) were considerably higher in rats who underwent CLP. In addition, PTL therapy inhibited the TNF-*α* (*p* ≤ 0.001), IL-1*β* (*p*=0.006), and IL-6 (*p*=0.002) expression levels induced by CLP. These findings provided molecular confirmation of the anti-inflammatory benefits of PTL.

**Figure 8:**
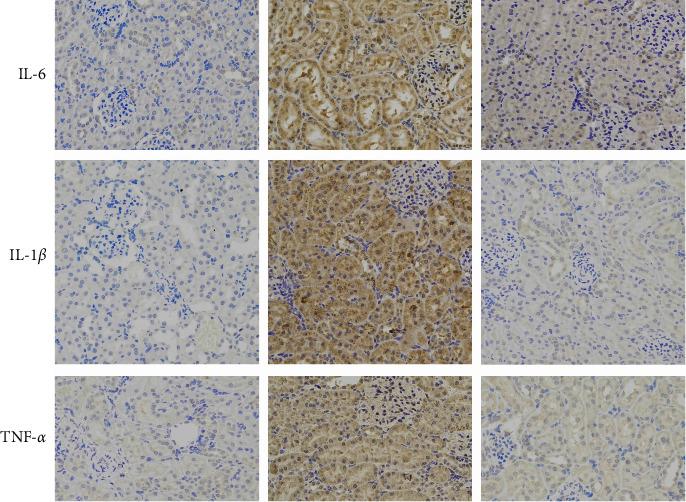
PTL inhibited CLP-induced cytokine expression in kidney tissues in vivo. In this study, we used immunohistochemical staining to evaluate IL-1*β*, IL-6, and TNF-*α* expression in kidney tissues. The findings shown here are typical of at least three individual trials. The 10 *μ*m scale bar shows the relative size of the images.

Then, the CLP-induced cytokines were evaluated via the immunohistochemistry technique. Interestingly, by comparison to the control and PTL groups, the CLP group showed significantly greater levels of positive IL-1*β*, TNF-*α*, and IL-6 expression. This result shows that inflammatory responses were detected after CLP treatment, leading to upregulated levels of inflammatory factors. However, inflammatory cytokine expression and the CLP-induced inflammatory response were both suppressed following PTL administration (Figure 8).

### 3.7. PTL Inhibited Activation of NF-*κ*B Signaling Pathway in Kidney of Septic Rats

**Figure 9:**
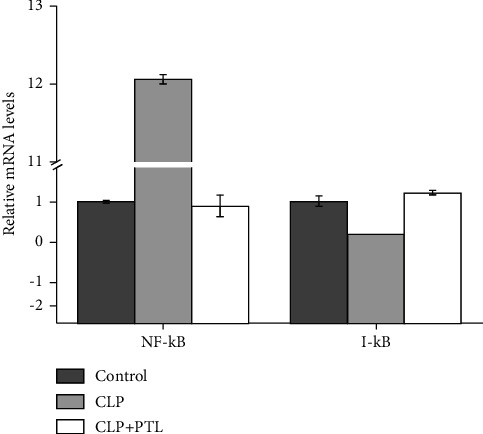
Expression of the NF-*κ*B-related genes with PTL treatment in the CLP model. The values are represented as mean ± SD from three independent samples.

**Figure 10:**
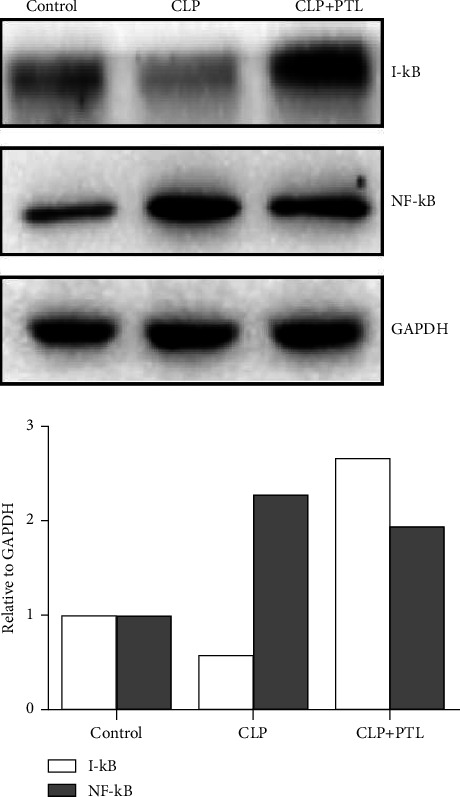
How PTL therapy prevented NF-*κ*B activation in the kidney tissues of rats with an infection. After the CLP procedure or the control operation, the rats received PTL treatment (control). The representative blots show the kidneys' levels of the proteins nuclear, NF-*κ*B, and I-*κ*B as measured by Western blot analysis.

PTL reduces NF-*κ*B expression and plays a crucial role in inflammation. Therefore, we further investigated the impact of PTL on NF-*κ*B activation in septic rats with the aim of better understanding the role of PTL in the renal inflammation response. We analyzed the protein expression of essential NF-*κ*B signaling pathway components with and without PTL therapy in a CLP-induced rat model. qPCR confirmed what was expected: the CLP drastically increased the NF-*κ*B and reduced the I-*κ*B protein, and PTL therapy showed a markedly inverse trend (Figure 9). The Western blot experiment confirmed what was expected: the CLP drastically increased the NF-*κ*B and reduced the I-*κ*B protein, and PTL therapy showed a markedly inverse trend (Figure 10). The obtained data revealed the potential role of the NF-*κ*B signaling pathway in the inflammatory response.

## 4. Discussion

Sepsis is the leading cause of admission to critical care units and is well recognized as a difficult clinical illness. Even though the complex pathophysiology of sepsis is complex, its early stages are marked by inflammation and its late stages by immunosuppression, which is associated with various abnormalities in cell death and coagulation reactions [23]. Previous studies have shown that the NF-*κ*B activation can enhance the transcription and increase the release of TNF-*α*, IL-1, and IL-2 [22, 28] improved the release of IL-6 and other inflammatory cytokines, which would further amplify the initial inflammatory signal, triggering, in this way, the “cascade reaction” of inflammatory mediators [23]. This reaction could damage the kidney and other organs and play a vital role in developing sepsis and renal damage. NF-*κ*B plays a role in sepsis by participating in mitochondrial apoptosis [29], cell senescence [30], and cell necrosis [31]. According to previous studies, PTL has been regarded as a specific and potent NF-*κ*B inhibitor that prevents the transcription and expression of NF-*κ*B. Moreover, it has also been reported to show anti-inflammatory, antiallergic, and antitumor effects [20, 32].

Here, we investigated the potential role of PTL in LPS-induced inflammation in glomerular mesangial cells. We found that upregulation of IL-1*β*, IL-6, and TNF-*α* levels was shown after LPS induction, and PTL reduced the inflammation levels induced by LPS. Further analysis showed that I-*κ*B phosphorylation was activated and enhanced the production of NF-*κ*B p65 in the LPS-induced group. As a result of PTL treatment, NF-*κ*B p65 expression was downregulated. These results suggested that PTL may play an important role in LPS-induced inflammation by regulating the NF-*κ*B signal pathway.

Then, we detected the protective impact of PTL in vivo using a rat septic model induced by the CLP technique. We found that PTL decreased the levels of IL-1*β*, TNF-*α*, and IL-6. The inflammatory markers IL-6, IL-10, and TNF-*α* are all strongly linked to the onset of sepsis, and as one of the most important inflammatory cytokines [33], TNF-*α* stimulates the production of IL-6 in endothelial cells [34]. TNF-*α* was also reported to have a major effect on inflammation, oxidative damage, and antiapoptosis. Therefore, our investigation revealed that PTL treatment attenuated the inflammation response of the kidney in septic rats.

Interestingly, these animals that received PTL intervention showcased, a significant reduction of inflammatory infiltration with a minor degree of injury. Our findings further showed that PTL may protect renal function by downregulating the levels of BUN, creatinine, KIM-1, and NGAL in the sepsis model. Furthermore, PTL therapy greatly increased the survival of septic rats induced by CLP operation.

We then investigated the influence of PTL on acute inflammation signaling pathways such as NF-*κ*B. In particular, recent research reported that kidney injury with inflammation was caused by the activation of the NF-*κ*B p65 pathway. In the cytoplasm, it was suppressed by I-*κ*B. In response to the rash-inducing stimulus, the I-*κ*B protein was phosphorylated by IKK and degraded through the ubiquitin-proteasome signaling pathway which resulted in the nuclear translocation of NF-*κ*B from its I-*κ*B binding site, where it functioned as a transcription factor for inflammatory genes [35]. Immunoblot analysis revealed that PTL therapy reduced NF-*κ*B p65 levels and increased I-*κ*B expression in septic kidneys as compared to the CLP group. Based on this observation, we hypothesize that PTL therapy may dampen the NF-*κ*B signaling pathway.

In conclusion, we demonstrated that PTL could regulate inflammatory factors in acute kidney injury and attenuate CLP-induced sepsis via the NF-*κ*B p65 signaling pathway. Therefore, PTL showed potential for playing a positive role in the treatment and prevention of sepsis. The verification of the specific molecular mechanism of PTL that regulates the NF-*κ*B signaling pathway represents the epicentre of our future studies.

## Data Availability

The data that support the findings of this study are available from the corresponding author upon reasonable request.
